# T166. SPATIAL INCOHERENCE OF LARGE-SCALE CORTICAL NETWORKS RELATES TO FORMAL THOUGHT DISORDER IN SCHIZOPHRENIA: A 7T MRI-BASED THICKNESS STUDY

**DOI:** 10.1093/schbul/sby016.442

**Published:** 2018-04-01

**Authors:** Lena Palaniyappan, Ali Al-Radaideh, Penny Gowland, Peter Liddle

**Affiliations:** 1 University of Western Ontario; 2 The Hashemite University; 3 University of Nottingham

## Abstract

**Background:**

The thickness of cerebral cortex varies across individuals as well as across different regions within an individual. Shared trophic or plastic influences such as repeated task-related recruitment of extant brain regions results in morphological covariance within large-scale brain networks. Pathological processes disrupting functional co-activation can result in higher than expected degree of variability within large-scale networks in an individual level, resulting in spatial incoherence. We studied spatial incoherence of cortical thickness in 17 cortical networks identified on the basis of well-known patterns of intrinsic connectivity, to identify the spatially incoherent networks and relate them to differences in severity of thought disorder among patients with schizophrenia.

**Methods:**

Ultra-high field 7T anatomical MRI scans (MPRAGE) were obtained from 20 subjects in a clinically stable, medicated early stage of schizophrenia, and 19 sex, parental socioeconomic-status and age matched healthy controls. Cortical thickness was estimated using Freesurfer v5.0, across 17 networks based on the parcellation scheme of Yeo et al. We computed within-network coefficient of variation in thickness (CVT) across vertices that constitute each network. Higher CVT of a network in a subject indicates higher spatial incoherence within the network for that individual. Independent 2-tailed t-tests were used to compare CVT of 17 networks between the 2 groups with FDR-corrected p=0.05 considered as statistically significant. We related CVT of affected networks to the scores of positive and negative Formal Thought Disorder measured using Thought and Language Index in patients.

**Results:**

Salience Network (aka Ventral Attention Network as per Yeo atlas), Default Mode Network and Central Executive Network (aka dorsal Attention Network in Yeo atlas) showed most significant reduction in MRI-derived cortical thickness (networks #8, #12, #15 as well as #16 of Yeo atlas). Only the Salience and Executive Networks (network #8 and #12) showed higher coefficient of variation in patients compared to controls, indicating either a failure of coordinated maturation or co-ordinated function. Higher spatial incoherence of Salience Network related to reduced mean thickness of Central Executive Network in patients with schizophrenia; this relationship was not seen in healthy controls (Fisher’s z test, p=0.02). Both higher coefficient of variation in Salience Network and lower mean thickness in Central Executive Network predicted the severity of positive but not negative thought disorder scores.

**Discussion:**

Our results indicate that (1) large-scale cortical networks involved in information processing (Salience and Executive Networks) show spatial incoherence in schizophrenia (2) the degree of spatial incoherence relates to the severity of disorganisation of thoughts and language in patients. Spatial incoherence may be the result of a dysmaturational or functional dysplastic effect reflecting inefficient cortical recruitment in schizophrenia. Within-subject morphological variability carries useful information that can potentially explain the elusive neural basis of complex symptoms such as formal thought disorder.

